# Supplementary material for: Neighbourhood socioeconomic disadvantage and body size in Australia’s capital cities: The contribution of obesogenic environments
Source: PLoS One. 2023 Jan 20;18(1):e0280223. doi: 10.1371/journal.pone.0280223 (PMC9858776; doi:10.1371/journal.pone.0280223)
Supplement: Attachment — Submitted filename: 20221006 Revise and Resublit NHSIII Rebuttal .docx [file pone.0280223.s001.docx]

We thank the Editor and Reviewer for their considered feedback on our manuscript. We very much appreciate the comments provided and the opportunity to improve our work. Please find below responses to each specific issue raised.

| **Comment ID** | **Reviewer Comment** | **Author’s Response** |
| --- | --- | --- |
| R1.1 | Introduction.  Page 5 opening paragraph. I think it is not appropriate to use “generalizability” in aims. For my understanding, study results can be generalized to a broader group of individuals with same characteristics. However, populations from two cities are geographically different, so study results from city A cannot be generalized to city B. Authors can think of a better word. Or just simply say to compare associations across cities. | We agree with the Reviewer that our use of the term ‘generalisable’ was inaccurate and inappropriate when describing the aims and focus of our study. In response, we have re-written the paragraph to address the Reviewer’s concerns (please see page 5, lines ~83-85 of the revised manuscript). |
| R1.2 | Methods.  Page 6 line 117-118: It is not so clear to me how the contextual variables were collected e.g., count of supermarkets and count of walkable amenities). Were GIS methods used? | Counts of supermarkets and walkable amenities within each network buffer were conducted by the ABS using ArcGIS Network Analyst. This information has now been added to the revised manuscript (please see page 11, lines 175 – 179). |
| R1.3 | Methods  Page 6. DAG. It is a little strange to include paths that are not tested in this study. Authors can think of highlighting the paths that are the focus of the present study. | In the DAG, we included all potential pathways between exposure and outcome to comprehensively, and accurately, depict how obesogenic environments are associated with body size. Our justification for this approach was described in a separate section about the DAG in the original manuscript. Despite this, the Reviewer has raised a good point about clearly identifying in the DAG the pathways that are examined (and not examined) in our study. As such, we modified the DAG by using solid boxes to indicate tested variables and dashed boxes to indicate untested variables (See updated DAG, separate file for insertion on page 8 of the revised manuscript). |
| R1.4 | Methods  It is not clear why authors used residential location as an indicator of obesogenic environment. Should cite some papers to support. In addition, density of/ access to fast food restaurants were widely used to measure obesogenic environment. Have authors considered using that? | It is now well established that within Australia’s capital cities residential areas located further from the central business district are less liveable and exhibit more of the built environment features of obesogenic neighbourhoods.^1^ Specifically, compared with urban neighbourhoods, suburban areas on the outskirts of Australia’s capital cities are more likely to be sprawling, and to have a built form that is less residentially dense, with a poorly interconnected street network, and a less diverse land use mix. These types of neighbourhoods are less walkable, they are often located a long way from employment and essential services, and they require greater reliance on private motor vehicles, and lower use of public transport and active travel (i.e., waking and cycling). In short, distant neighbourhoods are more likely to be obesogenic, and residents of neighbourhoods further from the city are more likely to be overweight or obese, as we have shown in our previous research.^2^ To address the Reviewer’s concerns we have now expanded our justification for why we used residential location as a marker for an obesogenic environment (please see page 10 lines 152-160 of the revised manuscript).   1. Giles-Corti B, Saghapour T, Turrell G, Gunn L, Both A, Lowe M, Roberts RJ, Butt A, Hooper P, Higgs C. Spatial and socioeconomic inequities in livability in Australia’s 21 largest cities: does city size matter? *Health and Place, 2022.* In Press. 2. Carroll SJ, Turrell G, Dale MJ, Daniel M. Residential location, commute distance, and body size: cross-sectional observational study of state and territory capital cities in Australia. *Journal of Transport and Health* 2021; 22:101122.   We agree with the Reviewer that density of, or access to, fast-food outlets is often used as a marker of an obesogenic environment. The NHS dataset included a measure of fast-food density (i.e., counts of fast-food stores in different sized buffers) and we explored the possibility of including this measure in our study. However, extensive preliminary analysis with the fast-food measure produced findings that were counterintuitive to what was expected, and to what had been published in previous research. Hence, we took a cautious and circumspect view of the measure and were not confident that it was producing accurate (i.e., valid, and reliable) results. Rather than persist with the measure and publish results that would have conflicted with and confused the field, we decided to take a conservative position and not include the measure in our study. |
| R1.5 | Methods  Page 7 line 127: individual factors influence neighborhood selection. It is possible that the association of disadvantaged neighborhoods and body size is spurious, i.e., the association is caused by a third “confounding” factor: individual-level socioeconomic status. How do authors rule out this possibility? | We agree with the Reviewer that neighbourhood self-selection based on individual-level socioeconomic factors might account for some of the association between neighbourhood disadvantage and body size. The NHS dataset that we used for our study did not include any specific measures of self-selection, hence we could not directly adjust for this factor in our models and so we cannot rule out the potentially confounding effect of unmeasured selection processes. However, as our study used multilevel analyses, we were able to adjust the association between disadvantage and body size for individual-level socioeconomic position (i.e., education, occupation, household income): thus, to some extent at least we were able to dampen the potential influence of self-selection into neighbourhoods based on one’s socioeconomic status. Further, the association between neighbourhood disadvantage and body size has been observed in many cross-sectional and longitudinal studies going back several decades and conducted across diverse countries, which provides a strong counter against the association being spurious. |
| R1.6 | Analytic Approach  I don’t’ understand why authors did not combine samples of 8 places and use the full sample to test 1) the association between neighborhood disadvantage and body size, and 2) contribution of obesogenic environments to this association. For these aims, I did not see a necessity of separating participants of 8 places.  The way of testing mediation effect of obesogenic environments did not tell you whether a mediation effect is significant or not. Sequence equation modelling (SEM) or generalized sequence equation modelling (GSEM) can do a greater job by testing the significance of mediator. Recommend authors consider SEM. | Previous international and Australian studies of obesogenic environments and overweight and obesity have primarily been conducted in one geographic context such as a single city or region, and the untested assumption is that findings pertaining to a particular city or region are generalisable to other (different) cities or regions. In this study, we test this assumption by performing separate analyses for each of Australia’s eight state and territory capital cities. In so doing, we examine whether and to what extent associations between neighbourhood disadvantage, obesogenic environments, and body size are the same or different across capital cities. If we had combined the samples from the eight places into one sample, we would not have been able to examine this question. In addition, Australia’s capital cities vary markedly on many sociodemographic, economic, and environmental characteristics that are likely to be important in shaping associations between neighbourhood disadvantage and body size^1^: combining all this heterogeneity into one sample would have obscured and distorted important information, increasing the likelihood of our modelled associations being attenuated to the null.   1. Carroll SJ, Dale MJ, Turrell G, Daniel M. Associations between supermarket availability and body size in Australia: a cross-sectional observational study comparing State and Territory capital cities. *BMC Public Health* 2021; 21:407 <https://doi.org/10.1186/s12889-021-10458-9>.   We agree with the Reviewer that if the purpose of our study was to test the mediating effects of obesogenic environments on the association between neighbourhood disadvantage and body size, then SEM would have been a more appropriate method. However, the analytic focus of our study was on the extent to which obesogenic environments accounted for variation between socioeconomically advantaged and disadvantaged neighbourhoods (grouped into quintiles) in their average levels of body mass index and waist circumference: for this purpose, our use of sequentially adjusted random coefficient (multilevel) models was appropriate. |
