# Supplementary material for: Neighbourhood socioeconomic disadvantage and body size in Australia’s capital cities: The contribution of obesogenic environments
Source: PLoS One. 2023 Jan 20;18(1):e0280223. doi: 10.1371/journal.pone.0280223 (PMC9858776; doi:10.1371/journal.pone.0280223)
Supplement: Attachment — Submitted filename: 20221215 Revise and Resubmit NHSIII Rebuttal.docx [file pone.0280223.s002.docx]

We thank the Editor and Reviewer for their considered feedback on our manuscript. We very much appreciate the comments provided and the opportunity to improve our work. Please find below responses to each specific issue raised.

| **Comment ID** | **Reviewer Comment** | **Author’s Response** |
| --- | --- | --- |
| R2.1 | Pg 13: The discussion of methods should include explicit mention of multilevel modelling approach, with the necessary details about units of analysis/ levels included. Currently, it isn’t explicitly clear what the authors mean by ‘accounting for clustering of individuals within SA1s”, and the term multilevel only appears subsequently in the paper. | In response to this comment, additional text is added at ~line 244-251, explicitly mentioning multi-level modelling and providing additional clarity around clustering – i.e., ‘A multi-level modelling approach was used with the unit of analysis at the level of the individual, adjusting for covariates and accounting for clustering of individuals within SA1’s. Clustering of participants within geographical location creates issues of non-independence due to correlations between measures. Multilevel models can account for this non-independence [51]. The first level modelled participant values of body size as a function of the obesogenic environment, while a second level accounted for spatial clustering within SA1’s, with a random intercept specified to allow for variations in values of body size across SA1’s.’ |
| R2.2 | In the introduction, the authors highlight that they were “documenting the association between neighbourhood disadvantage and body size and examining the contribution of obesogenic environments to this association.” The choice of word ‘contribution’ here suggests that the authors are looking at how obesogenic environment are along the causal pathway between neighborhood disadvantage and body size—i.e., how it acts as a mediator. The DAG also suggests that the authors are indeed treating the obesogenic environmental characteristics as mediators as they are visually represented as being part of the causal pathway.  Yet at the same time, some of the discussion within the paper seems to suggest that the authors are seeing obesogenic environments are potential confounders instead. For instance, in the discussion about why distance from CBD might relate to neighborhood disadvantage and BMI/waist circumference (pg 10 tracked changes version), the authors seem to be suggesting that other factors related to distance to CBD (e.g. urban sprawl, public transport usage) which are co-occurring with neighborhood disadvantage BUT not directly attributable/ related to it might be driving the association. In other words, they seem to be describing ‘distance to CBD’ as a confounder.  Are the authors looking at obesogenic environments as potential mediators or confounders of the relationship between neighborhood disadvantage and body size? This distinction should be made clear up front and be consistently applied throughout the paper, as this would affect how their findings should be interpreted. | As depicted in the DAG, distance to the CBD is conceptualised as a factor that might help explain differences between advantaged and disadvantaged neighbourhoods in their BMI and waist circumference: in our study, distance to the CBD is NOT conceptualised as a confounder. A key question that we examine is whether higher levels of body size in disadvantaged neighbourhoods are partly due to these areas being distal from the CBD and hence more likely to have built environments that are more sprawling, less walkable, with poorer public transport networks and greater dependence on private motor vehicles. Importantly, we find support for our conceptualisation of distance to the CBD as an explanatory factor: in several capital cities, the magnitude of the association between neighbourhood disadvantage and body size attenuates noticeably after adjustment for distance, strongly suggesting that residents of disadvantaged and advantaged have worse and better access respectively to environments that are conducive to healthy bodyweights. |
| R2.3 | I find the choice of term ‘residential location’ rather unintuitive. Why not just call it as it is, ‘distance to CBD’ ? | We agree with the Reviewer about the term ‘residential location’ being unintuitive. In response, we have changed the term to ‘residential distance to the CBD’ throughout the manuscript. |
| R2.4 | Additionally I am not entirely clear whether ‘residential location’ as defined in this paper is meant to be an ‘obesogenic environment’ characteristics being tested, or more of a control variable. My read is that the authors are treating it as the former. If so, I question the value of having such a broad ‘obesogenic environmental characteristic’ which is actually more of a proxy for many other more specific characteristics that the authors themselves highlight (pg 10 tracked changes version, where they discuss urban sprawl; use of public transport; other facets of walkability that are correlated with distance to CBD) that might explain why distance to CBD might be related to neighborhood disadvantage as well as obesity. As a ‘catch-all’ measure, it is unable to elucidate which aspects of the environment is actually obesogenic and thus needs to be addressed through urban design or policy…since ‘distance to CBD’ is not a environmental characteristic that can be changed! I would question the value of treating ‘distance to CBD’ as an obesogenic environmental characteristic. | Previous Australian research has shown that socioeconomically disadvantaged neighbourhoods are more likely to be located distal from each capital city’s CBD, and that these neighbourhoods are less likely to have built environments that are conducive to a healthy body size. For example, distal (and often lower SES) neighbourhoods are more likely to be sprawling and have a built environment that is less residentially dense, with a poorly interconnected street network, and a less diverse land use mix. Consequently, these same neighbourhoods are less walkable, they are often located a long way from employment and essential services, they require a greater reliance on private motor vehicles and are less conducive to the use of public transport and active travel (i.e., walking and cycling). It is these (and related) characteristics of a distal neighbourhood’s built environment that can be considered ‘obesogenic’, and its arguably for these same reasons why residents of these areas are more likely to overweight and/or obese. In our study, we used ‘residential distance to the CBD’ as a broad proxy for an obesogenic environment. As the Reviewer correctly notes, this is less than ideal, and the use of specific obesogenic measures would have been preferred. However, such measures were not available to us, and we were required instead to use a general non-specific measure of an obesogenic environment. This limitation notwithstanding, our measure of residential distance to the CBD was associated with neighbourhood disadvantage, and it accounted for some of the variation between advantaged and disadvantaged neighbourhoods in their BMI and waist circumference. Given this, we are strongly of the view that that our proxy distance measure, despite its limitations, was capturing some unmeasured aspects of an obesogenic environment, thus supporting its inclusion in our study. Further, to address the Reviewer’s concerns we have included text in the revised manuscript that notes the limitations of using distance to the CBD as a proxy measure of an obesogenic environment (see lines 482 – 492). |
| R2.5 | Could the authors include a better synthesis of the results from the models looking at BMI versus waist circumference as a measure of adiposity? I found it hard to follow the current discussion—could it be clearer where results differ / concur between these two measures? | As suggested by the Reviewer, we better synthesised where possible the results from models that examined BMI and waist circumference. On re-reading the Discussion, we now believe the text is clearer and easier to follow for the reader. |
| R2.6 | The authors argue that a major contribution of this paper is in its testing of the relationship of interest across different cities. To this end, I think the paper needs more introduction to the cities being studied ,especially for readers unfamiliar with Australia. Furthermore, is there a working hypothesis upfront why certain cities may exhibit certain patterns versus others? This is discussed briefly in the ‘Discussion’ section, but could the authors consider adding some in the Introduction to give clearer grounding for this particular piece of the analysis. | The Reviewer’s point is well made: readers unfamiliar with Australian cities may receive insufficient orientation to the context from our Introduction. In response to the Reviewer’s concerns, we have added the following text at ~lines 70-74 : ‘Australian capital cities are generally conceived of as mono-centric, anchoring on a central business district [34, 35], with low levels of suburban housing density [36] resulting in urban spawl and peripheral or distal suburbs substantially distant from, and different to, inner city suburbs in terms of access to amenities, resulting in spatially patterned inequity [36]. Lower levels of liveability are likewise evident in distal suburbs in Australian capitals [37, 38].’ |
| R2.7 | What is an ‘SA1’? What size area does it cover and what’s its population size? | Lines 158 – 164 of the revised manuscript provide a definition and description of an SA1. As part of this text, we report population sizes for SA1s across Australia and in metropolitan cities: however, we were not able to report area-sizes as these data are not publicly available. |
